# Research on Experimental Validation and Prevention Strategies for Pin Shaft Failure in Concrete Pump Trucks

**DOI:** 10.3390/s25216518

**Published:** 2025-10-22

**Authors:** Wuhe Sun, Kai Cheng, Bowen Guan, Bin Wu, Erfei Zhao

**Affiliations:** School of Mechanical and Aerospace Engineering, Jilin University, Changchun 130022, China; sunwh20@mails.jlu.edu.cn (W.S.);

**Keywords:** accident prevention, equivalent experiment, high-stress zone, pin shaft failure, stress simulation

## Abstract

This study focuses on the pin shaft failure accidents occurring during the construction of concrete pump trucks and hypothesizes that the accidents are caused by improper installation of the pin shaft mounting angle (defined as the angle between the oil passage axis and the horizontal plane). First, the actual operating conditions were simplified to design an equivalent test, through which the stress distribution of the pin shaft under the 360° rotation condition was measured and understood. Then, simulation analysis was conducted to verify the stress concentration phenomenon under different pin shaft mounting angles. The results show that the pin shaft mounting angle at the accident site falls within the high-stress zone centered on the oil cylinder axis, verifying the hypothesis. In addition, the high-stress zone of the pin shaft does not change with the rotation angle of the pin shaft; it is only related to the position of the oil cylinder axis and distributed symmetrically around the oil cylinder axis. Therefore, to prevent the pin shaft failure accidents, the mounting angle of the pin shaft can be adjusted to keep it away from the high-stress zone near the oil cylinder axis.

## 1. Introduction

**Figure 1:**
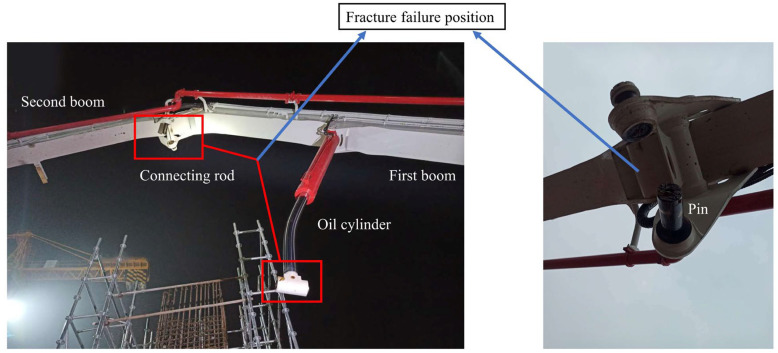
Concrete pump truck boom arm pin (first boom and connecting rod) on-site damage diagram.

Concrete pump trucks play a crucial role in modern construction projects, delivering concrete to designated locations through their pumping systems, thereby significantly improving construction efficiency [1,2,3]. However, during long-duration, high-intensity operations, the reliability and durability of pump trucks’ key components often face challenges. Particularly, the pin shaft connecting the connecting rod and cylinder is a critical component; its failure can lead to the inability of the pump truck to operate normally, severely impacting construction progress and safety [4,5]. This paper studies a pin shaft failure accident case on a 65-m six-section boom concrete pump truck (Figure 1).

**Figure 2:**
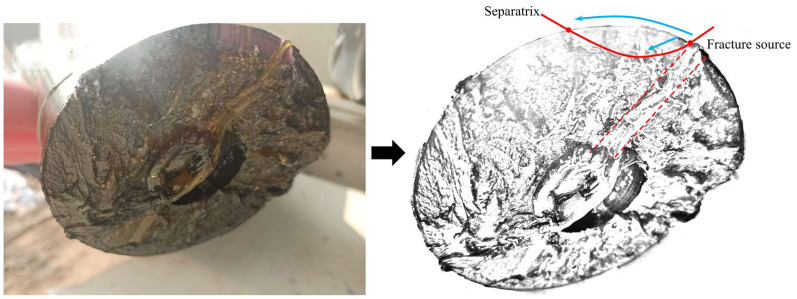
Cross-sectional view of the pin shaft. (The red line marks the boundary between the smooth area and the rough area. The blue line indicates the propagation direction of the crack. The dashed line represents the oil passage.)

**Figure 3:**
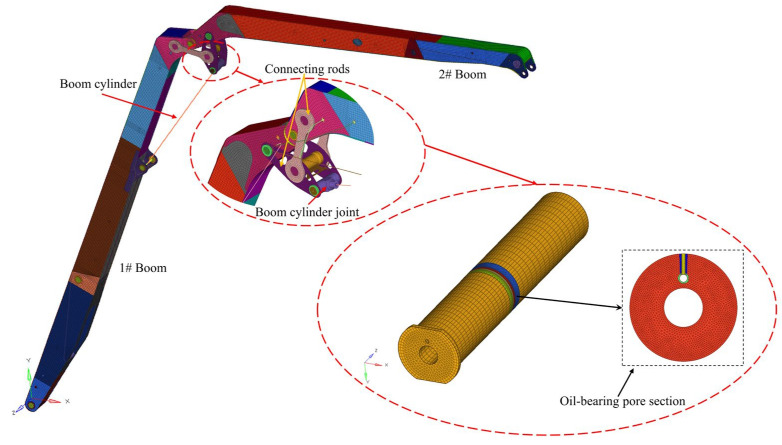
Localized finite element model of the accident site under operating conditions. (The red circle is an enlarged view of the local structure).

Based on the image analysis of the pin shaft failure at the construction site (Figure 2), it is hypothesized that the pin shaft failure might be caused by improper installation angle. Specifically, when the oil hole axis of the pin shaft aligns with the axis of the boring cylinder (Figure 3), this installation exposes the oil hole directly to the high-stress zone. Due to the significant stress concentration effect at the oil passage opening, this situation negatively impacts the structural integrity of the pin shaft and amplifies the stress peak in the stress concentration zones [6,7], leading to the pin shaft failure.

To verify this hypothesis, this study conducts an equivalent test replicating the accident site operating conditions. The pin shaft installation angle is rotated from 0° to 360° (in increments of 10°) to evaluate the variation in the maximum stress at the oil passage hole across the full range of motion. To ensure the accuracy and validity of the results, numerical simulation is performed under identical operating conditions. This research will reveal how the pin shaft installation angle affects stress concentration at the oil hole and the relationship between the high-stress zone and the boring cylinder axis position.

Although the pin shaft failure significantly impacts the safe operation and reliability of the pump truck, research on the causes of failure in pump truck boom pin shafts remains a blank area in both domestic and international studies. This study proposes an innovative hypothesis, providing new theoretical basis and practical guidance for understanding the failure mechanism of boom pin shafts. Through this research, a novel method is introduced for preventing the pin shaft failures in similar construction machinery, thereby filling the research gap in this field.

## 2. Materials and Methods

### 2.1. Problem Formulation

According to feedback from on-site personnel, the pin shaft did not last long from emitting audible noise to failure. The failure cross-section of the pin shaft is shown in Figure 2, which indicates that the failure originated at the edge of the oil passage hole. The main cause of this failure was the excessive stress at the oil passage hole [8,9,10]. Based on the location of the failure [11,12,13] and the fracture appearance, it can be determined as fatigue failure [14,15,16].

**Figure 4:**
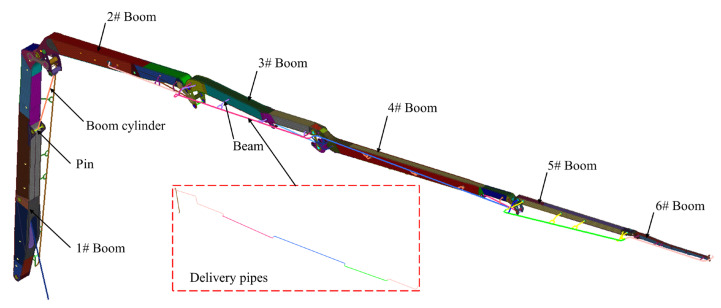
Finite element model of the accident site under operating conditions.

**Figure 5:**
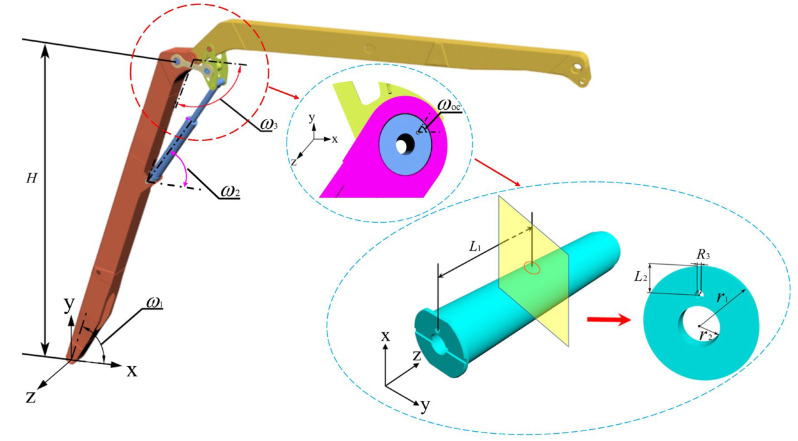
Schematic diagram of the geometric model dimensions at the accident site. (The red/blue circle is an enlarged view of the local structure).

In actual engineering applications, although incidents of this nature are relatively rare, their consequences, once they occur, can be catastrophic. The boom configuration during the accident is shown in Figure 4, where one boom is at a 60° angle to the horizontal, and the second to sixth booms are fully extended horizontally. Figure 3 illustrates the connection between the cylinder and connecting rod via the pin shaft, with the oil passage hole mesh refined and the oil passage cross-section diagram provided. Figure 5 displays other structural parameters related to the accident site conditions, with structural parameters detailed in Table 1 and Table 2.

During the service life of concrete pump trucks, the pin shafts are subjected to cyclic loading [17,18,19,20] and are influenced by equipment vibration effects, making them prone to failure due to stress concentration accumulation at one end. Once the pin shaft fails, it directly causes the connected hydraulic cylinder to deviate from its intended installation position, compromising the original load transmission path and structural stability. Ultimately, this triggers abnormal functionality in the boom assembly, preventing normal operational movements.

### 2.2. Principle and Purpose of the Experiment

**Figure 6:**
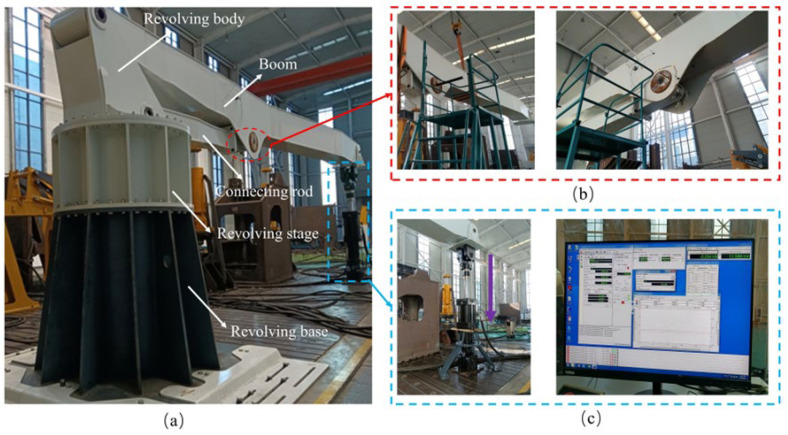
Test bench construction. (**a**) Overall test bench; (**b**) Test pin shaft; (**c**) Loading system and data acquisition system.

During the actual operation of concrete pump trucks, the loads acting on the pin shaft are primarily from the weight of the front-end structures [21]. To simulate the load performance under real operating conditions, the test adopts a test bench structure, as shown in Figure 6. The main components of the test bench include the base, turntable, boom, and connecting rod, connected via pin shafts among the turntable, boom, connecting rod, and loading fixture. The design of the test bench not only considers structural stability but also aims to replicate the load-bearing conditions of the pin shaft as closely as possible under real-world scenarios. During the test, the boom is a genuine folded boom structure (3# Boom of Figure 4). The base is fixed on the platform, and the turntable is rigidly connected to the lower part of the base via bolts.

The primary objective of this test is to study the stress distribution of the pin shaft and understand its stress variation patterns under different installation angles. Specifically, the test aims to reveal the stress distribution at the oil passage hole of the pin shaft as the installation angle changes, as well as the stress distribution relationship between the pin shaft installation angle and the cylinder axis. This test employs the DHDAS dynamic signal acquisition and analysis system. Strain gauges are adhered to the oil passage hole openings of the pin shaft to obtain stress distribution data under different load conditions and installation angles [22]. Load is applied vertically downward by an actuator (maximum capacity 1000 kN) fixed to the platform, simulating the load conditions experienced by the pin shaft during actual operation. Strain gauges continuously collect stress data from the pin shaft under various conditions. By comparing the experimental data with finite element calculation values, the accuracy and computational precision of the finite element model are verified. This not only aids in refining the finite element model and enhancing its ability to simulate real-world operating conditions but also provides significant guidance for the design and installation of the pin shafts in practical engineering applications.

### 2.3. Test Subjects and Equipment

The subject of this test is the pin shaft. To ensure the reliability and accuracy of the test results, three pin shafts are used in the experiment. Each pin shaft undergoes the same preliminary treatment (The cylindrical surface of the pin is in good condition. Manufactured by the equipment manufacturer in accordance with actual technical requirements, no visible scratches or other surface defects were found upon inspection.) and installation procedure to ensure consistency in the loads and conditions experienced during the test.

**Figure 7:**
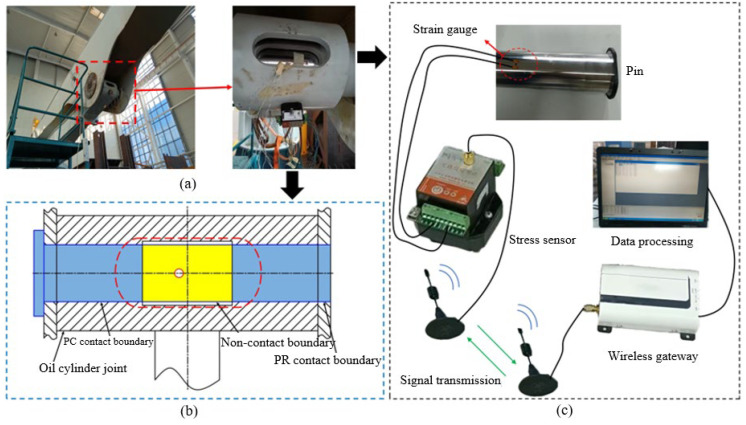
Experimental equipment diagram. (**a**) Strain gauge and test model system; (**b**) Pin shaft installation system; (**c**) Signal transmission system.

The test equipment includes strain gauges, stress sensors, signal transmission systems, and data processing systems. These components collectively form a complete experimental testing platform for measuring and analyzing the stress variations in the pin shafts under different operating conditions. Based on the characteristics of the pin shaft failure and the direction of bending deformation, the strain gauges are strategically placed at the upper and lower surfaces of the oil passage hole on the pin shaft, oriented along the shaft’s axis, as shown in Figure 7a,b. This arrangement ensures accurate and effective real-time monitoring of stress results during the loading process. To achieve real-time data monitoring and transmission, the strain gauges are connected to the stress sensors via wires [23]. The stress sensors convert the stress signals detected by the strain gauges into transmissible electrical signals, which are then transmitted via a wireless system to the data processing system. The complete signal transmission process is illustrated in Figure 7c. The data processing system consists of a computer and specialized software, designed specifically for receiving, storing, and analyzing signals transmitted from the stress sensors. It enables real-time display of stress variations in the pin shaft, processes and analyzes the acquired data, and selects the maximum stress value as the experimental result.

### 2.4. Test Method

**Figure 8:**
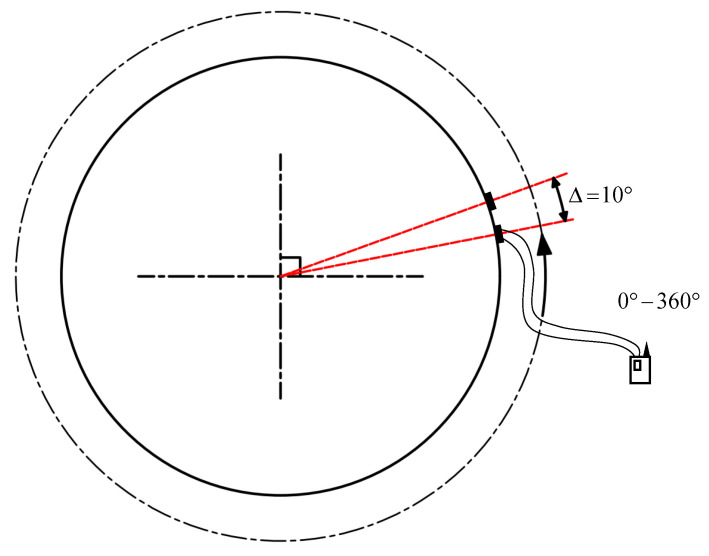
Strain gauge 360° rotation diagram.

First, assemble all structural components. On both the boom side plate and the pin shaft surface, mark reference points for angle alignment. During the installation of the pin shaft, ensure precise alignment with these reference points. The initial installation angle of the oil passage hole is set to 0° (the oil passage hole axis forms a 0° angle with the +X axis). Considering that excessive loads can lead to shaft bending deformation, thereby increasing the difficulty of installation angle adjustment, a staggered loading scheme is designed: the load is incrementally increased from 0 kN to 100 kN (multiple pre-loading cycles are performed to ensure that the stress at the oil passage hole does not exceed the material yield strength of 650 MPa under a 100 kN load), and then slowly reduced to 0 kN. This loading cycle is repeated three times to ensure data reliability. During the loading process, stress data monitored by the strain gauges are collected in real-time through the stress sensors and signal transmission system. Subsequently, the installation angle of the pin shaft is sequentially adjusted to 10°, 20°, and so on, up to 360° (the rotation direction is shown in Figure 8). After each angle adjustment, the aforementioned loading and data acquisition process is repeated. After completing the tests for the first pin shaft, it is removed, and the entire testing procedure is repeated for the second and third pin shafts in sequence until all test specimens have been fully tested.

### 2.5. Simulation Model

This stress analysis is conducted based on physical field principles. During modeling, it is assumed that the material exhibits a linear stress–strain relationship up to the yield point. According to the technical requirements of the equipment manufacturer, the test pin shaft undergoes heat treatment, significantly increasing its yield strength above 650 MPa (Table 3); however, in finite element calculations, the properties of the shaft material without heat treatment are intentionally used, incorporating material property deviations to retain a safety factor and ensure the engineering safety of the analysis results. The von Mises stress formula is given by:

(1) $$\sigma_{vm}=\sqrt{\frac{1}{2}\left[{\left(\sigma_{1}-\sigma_{2}\right)}^{2}+{\left(\sigma_{2}-\sigma_{3}\right)}^{2}+{\left(\sigma_{3}-\sigma_{1}\right)}^{2}\right]}$$

Herein, $\sigma_{1}$, $\sigma_{2}$ and $\sigma_{3}$, respectively, represent the first, second, and third principal stresses in the spatial context.

**Table 3:** Material properties of the pin.

Material Property	42CrMo
Density (Kg/m^3^)	7850
Young’s Modulus (GPa)	212
Poisson’s Ratio	0.28
Elastic Limit (MPa)	489
Tensile Yield Stress (MPa)	650
Ultimate Tensile Stress (MPa)	1000
Fracture Toughness (MPa·m^1/2^)	50–80
Hardness	HB 220–230
Elongation (%)	12

**Figure 9:**
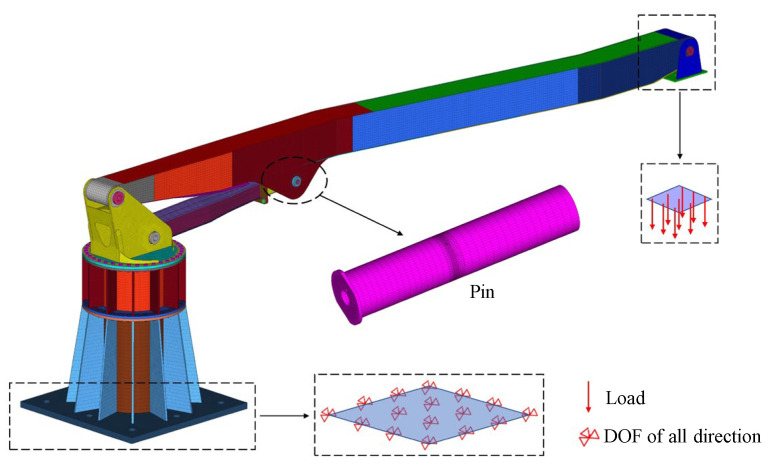
Finite element model of experimental conditions.

In the finite element model, the pin shaft and bushing are modeled using SOLID185 solid elements (as shown in Figure 9). SOLID185 is a type of three-dimensional solid element suitable for simulating complex geometries and material properties. During modeling, to capture variations in stress concentration zones, mesh refinement is specifically applied to the cross-section containing the oil hole [24]. Calculations were performed using models with grid sizes of 2 mm, 3 mm, 4 mm, 5 mm, and 6 mm. The results indicate that the maximum stress concentration values at the oil hole differ by no more than 1.6% across these models. Considering the increased computational load from excessive mesh density, the final analysis adopted a 4 mm grid size [25,26].

The boom and connecting rod are simulated using SHELL181 shell elements, which are suitable for thin-walled structures and effectively model the mechanical behavior of box-type structures welded from plates under actual operating conditions. To accurately simulate the force transmission between the pin shaft and bushing, contact element pairs (CON173 contact elements and TAG170 target elements) are employed to model the nonlinear behavior of contact force transmission [27]. The turntable and components below it are simulated using SOLID185 solid elements. The entire finite element model, as shown in Figure 9, consists of 325,949 elements.

### 2.6. Material Attribute Type

In this study, the pin shaft is made of 42CrMo steel. The material properties are shown in Table 3 [28].

### 2.7. Boundary Conditions

**Figure 10:**
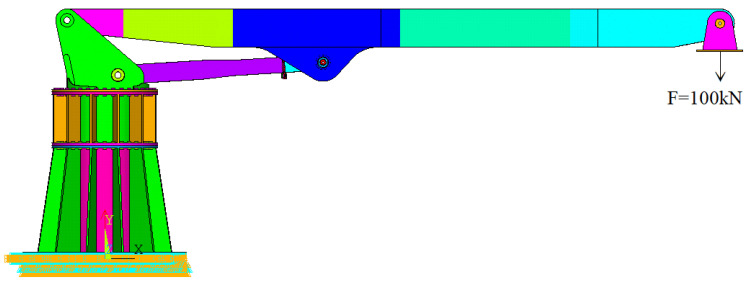
Constraints and load diagrams.

In this simulation model, displacement boundary conditions are applied to the base bottom (located at the lower left of Figure 10) to ensure the stability and immovability of the base during loading. The boom, turntable, connecting rod, and load application fixture are connected through three-dimensional solid pin contact settings, ensuring rotational freedom between all structural components.

In this simulation model, load boundary conditions are applied to the bottom of the connection fixture, imposing a 100 kN downward vertical load (as shown on the right side of Figure 10). This load is transmitted through the connection fixture to the pin shaft, simulating the maximum load state that the pin shaft experiences during actual operation. At this point, the horizontal inclination angle of the cylinder axis is 3.515°.

## 3. Results

In the processing of the pin shaft test data, the arithmetic mean method is employed. The test average values and results at various angles are presented in Table 4, while the calculated values and corresponding results at different angles are shown in Table 5.

### 3.1. The Pin Shaft 360° Rotation Test Results

As shown in Table 4, the black bold numbers represent high-stress data with stress values ≥ 290 MPa. This threshold is determined based on the equipment manufacturer’s requirements, where 290 MPa is derived from dividing the material yield strength of 650 MPa by a safety factor of 2.25 (i.e., 650 MPa/2.25). Analysis of the relationship between the pin shaft installation angle and corresponding stress test values within the range of 0° to 360° reveals periodic fluctuations with overall symmetry. The stress distribution exhibits a specific pattern characterized by “a single primary minimum, two secondary minima, and symmetric high values at the start and end angles.”

(1) Core Low Valley Intervals: The intervals at 90° (62.52 MPa) and 260° (74.31 MPa) exhibit significantly lower test values. At 90°, the stress reaches its minimum value within the full cycle, showing an 89% reduction compared to 0° (583.09 MPa). The 260° point represents a secondary low valley, with slightly higher values than at 90°. These two points constitute the key low response points within the cycle.

(2) High Value and Rising Interval: The test values at 0° (583.09 MPa) and 350° (577.81 MPa) are nearly identical, constituting the high-value ends of the cycle and demonstrating good cycle closure. After 90°, the test value gradually increases, reaching its first relative high value at 170° (478.15 MPa). The stress remains at a high level between 180° and 190° (465 MPa). After 260°, the stress rises again, returning to the high-value interval between 340° and 350°.

(3) Critical Change Stages: The interval from 0° to 90° is characterized by a continuous and rapid decrease in test values, dropping from 583.09 MPa to 62.52 MPa. This represents the stage with the largest reduction and most significant changes within the entire cycle. From 90° to 260°, the test values exhibit an “increase-decrease” fluctuation pattern, followed by a continuous rise after 260°. The overall trend of changes demonstrates a correlation with the angular cycle.

### 3.2. The Pin Shaft 360° Rotation Calculation Results

As shown in Table 5, the calculated values also exhibit periodic fluctuations with angle changes, oscillating alternately above and below a certain range overall within the 0° to 360° range. This distribution pattern is characterized by “two low valleys and multiple peaks.”

(1) Low Valley Intervals: The calculated values reach their lowest points near 90° (61.59 MPa) and 260° (76.74 MPa). Among these, the value at 90° represents the absolute minimum within the entire cycle, decreasing by approximately 90% compared to the value at 0° (602.82 MPa).

(2) Peak Intervals: The highest test values occur near 0° (602.82 MPa) and 350° (604.89 MPa), with values close to each other, forming the high-value endpoints of the cycle. Additionally, relatively high secondary values appear at angles such as 170° (495.97 MPa), 190° (487.28 MPa), and 320° (482.25 MPa).

(3) Change Magnitude: During the period from 0° to 90°, the calculated values show a continuous and rapid decrease, dropping from 602.82 MPa to 61.59 MPa, representing the largest drop. After 90°, the values gradually rise, falling back to 347.9 MPa at 180°. Subsequent changes involve fluctuations with rises and drops, and by 350°, the values return to the high-value interval, completing a cyclic pattern with the data at 0°.

Commonalities and Differences in the Variation Patterns of Measured and Calculated Values for the Pin Shaft from 0° to 360°:

(1) Both parties exhibit common patterns: periodicity and alignment with core change trends.

(1) Prominent Period Closure Characteristics: Both test values and calculated values exhibit periodicity from 0° to 360°, with “corresponding high values at the beginning and end” forming a cycle. The numerical values at the start and end of the cycle are very close—test values at 0° and 350° are 583.09 and 577.81, respectively, with a difference of only 5.28; calculated values at 0° and 350° are 602.82 and 604.89, respectively, with a difference of only 2.07. Both test and calculated values achieve a “high-value start to high-value end” cycle closure, demonstrating their alignment with the angle period.
(2) Consistent Core Low Valley Interval: Both test and calculated values reach their lowest point across the entire cycle at 90°, with a secondary low valley appearing near 260°. Specifically, the test values show a minimum of 62.52 at 90° and a secondary low of 74.31 at approximately 260°, while the calculated values reach 61.59 at 90° and 76.74 at approximately 260°. The angular positions of both the minimum value and the secondary low valley are identical, indicating that both sets of data strongly agree on the weakest mechanical response occurring at 90° and 260° for the pin shaft.
(3) Synchronized Critical Change Phases: Both test and calculated values exhibit three distinct phases across the full cycle: a rapid decline from 0° to 90° (which constitutes the largest drop interval for both, with test values decreasing by 89% and calculated values by 89.8%), followed by a period of oscillatory fluctuations characterized by multiple peaks and valleys from 90° to 260°, and finally a steady rise from 260° to 350°. The overall temporal evolution pattern of these changes demonstrates complete synchronization between the test and calculated data.

(2) Characteristics of differences between the two: the magnitude of numerical values differs and the amplitude of local fluctuations differs.

(1) Difference in Overall Numerical Levels: The calculated values consistently exceed the test values throughout the entire cycle, with the difference remaining relatively stable. At 0°, the calculated value (602.82) is 19.73 higher than the test value (583.09); at 350°, the calculated value (604.89) is 27.08 higher than the test value (577.81). On average, the calculated values are approximately 20~30 MPa higher than the test values across the full cycle. This discrepancy is likely attributed to errors inherent in numerical simulation versus experimental testing, such as assumptions in material parameters or interference from testing environments.
(2) Differences in Local Fluctuation Magnitude: Within the 150–190° interval, the calculated values exhibit greater fluctuation amplitude compared to the test values. Specifically, the calculated values rise sharply from 322.36 at 150° to 495.97 at 170° (an increase of 53.8%) and then drop to 347.90 at 180° (a decrease of 30%), whereas the test values increase from 404.96 at 150° to 478.15 at 170° (an increase of 18.1%) and then decrease slightly to 465.82 at 180° (a decrease of 2.6%). This suggests that the calculated values display more pronounced “steep rises and steep drops,” while the test values show relatively smoother local changes, which may likely be related to the simplifications in the simulation model versus the smoothing effects inherent in experimental data.

### 3.3. Calculation Results for the Pin Shaft at 0° Installation Angle

**Figure 11:**
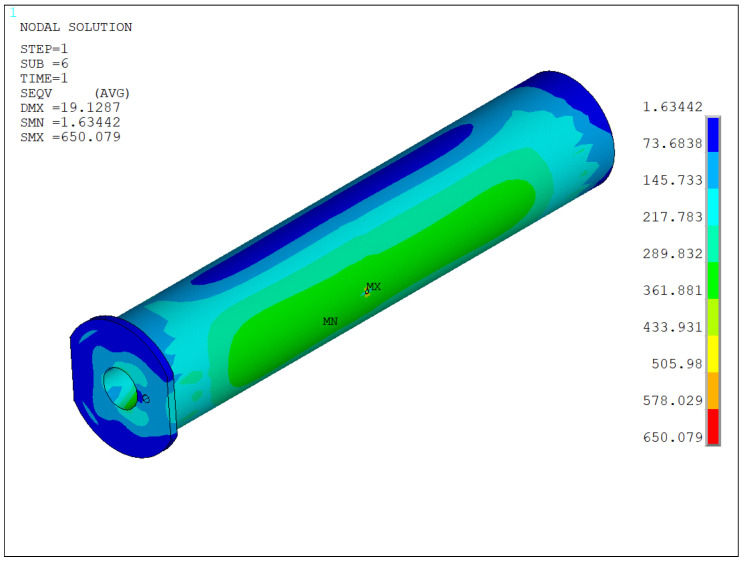
Von-Mises stress contour of the pin shaft structure at 0° installation angle.

When the installation angle of the pin hole is set to 0°, the overall stress distribution characteristics of the structure can be intuitively presented through the stress cloud diagram shown in Figure 11. From the color gradient and numerical annotations of the stress cloud diagram, it can be observed that under this condition, the deformation mode of the pin structure is primarily bending deformation, with no significant torsional or shear deformation occurring. Additionally, the stress distribution exhibits a pronounced localized concentration phenomenon, and the area of maximum stress is precisely located at the edge of the oil hole. Due to the structural discontinuity of the oil hole, a stress concentration effect occurs at this location. According to the data obtained from the stress cloud diagram and calculations, the maximum stress value at this point reaches 650 MPa. This area needs particular attention to ensure that the structural strength meets the design service requirements.

**Figure 12:**
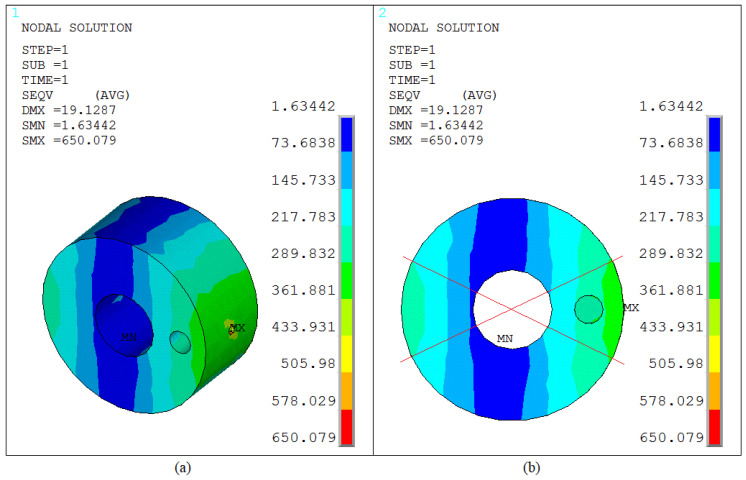
Von-Mises stress contour of the oil channel structure at 0° installation angle. (**a**) Von-Mises stress contour of the oil hole; (**b**) Von Mises stress distribution on the cross-section of the oil hole.

The structural cross-sectional stress distribution characteristics at the pin oil hole can be clearly observed through the cross-sectional stress cloud diagram shown in Figure 12a. From the stress gradient and regional divisions in the cloud diagram, it is evident that stress concentration in the pin primarily occurs in the area surrounding the oil hole. The location of the maximum stress value is precisely positioned in the outer edge region around the oil hole. This area experiences a significant stress concentration effect due to the structural discontinuity caused by the oil hole opening, which disrupts the stress transmission path. Further analysis of the high-stress zones (stress values ≥ 290 MPa), combined with angular coordinate analysis in Figure 12b, reveals that their distribution is concentrated within three continuous angular intervals: [0° to 30°], [150° to 210°], and [330° to 360°]. This provides a clear basis for stress-controlled regions, offering a solid foundation for targeted adjustments to the structure surrounding the oil hole in subsequent design or analysis phases.

## 4. Discussion

### 4.1. Discussion on Results for the Pin Shaft at 0° Installation Angle

(1) When the installation angle of the pin oil hole is set to 0°, the overall stress distribution characteristics of the structure can be intuitively presented through the stress cloud diagram shown in Figure 11. From the stress gradient and deformation trends in the cloud diagram, it is evident that under this condition, the pin structure undergoes primarily bending deformation. The geometric center of the oil hole aligns with the midpoint of the pin axis, positioning it at a critical load-bearing area. Stress analysis results indicate that the maximum stress in the pin is concentrated in the oil hole region. This is attributed to the abrupt change in cross-sectional dimensions caused by the oil hole opening, which disrupts the stress transmission path and leads to localized stress concentration. According to data extraction, the maximum stress value reaches 650 MPa. Further observation reveals that the high-stress zones (stress values ≥ 290 MPa) exhibit continuous distribution along the radial direction (perpendicular to the oil hole axis) and circumferential direction (around the oil hole circumference), thereby identifying the core control regions for subsequent structural strength adjustments.

(2) The stress distribution characteristics at the pin oil hole can be clearly characterized through the stress cloud diagram shown in Figure 12. From the numerical annotations and color gradients in the cloud diagram, it is evident that stress concentration in the pin primarily occurs in the region surrounding the oil hole, with the maximum stress precisely located in the outer edge area around the oil hole. This area experiences a significant local stress concentration effect due to the abrupt change in the structural cross-section caused by the oil hole opening, which disrupts the continuity of stress transmission. Further analysis of the high-stress zones (stress values ≥ 290 MPa), combined with coordinate mapping of the stress cloud diagram, reveals that their distribution is concentrated within three continuous angular intervals: [0° to 30°], [150° to 210°], and [330° to 360°]. Given the geometric characteristics of the pin (cross-section weakening due to the opening structure), the persistent stress concentration at the oil hole region may serve as the initiation and propagation site for fatigue cracks. This analysis conclusion aligns closely with the actual failure location observed in Figure 5, validating the rationality of the association between stress concentration and structural failure.

(3) As shown in Figure 12, the stress distribution in the pin exhibits a significant gradient variation characteristic: the high-stress zone centered on the oil hole gradually attenuates as the distance from the oil hole center increases, transitioning to a low-stress zone. Overall, the stress field distribution follows the pattern of “core concentration and outward diffusion.” Further localization analysis of the high-stress zone (specifically the load transmission area in contact with the cylinder sleeve) reveals that it primarily concentrates at the two ends of the pin horizontally. When connecting the high-stress zones at both ends, the angle between the connecting line and the cylinder axis is approximately 3.515°, with the directions largely consistent. The essence of this phenomenon lies in the load transmission path: the cylinder axis direction serves as the primary force transmission direction. When loads are transmitted along this direction to the pin, it causes the pin to experience greater forces in the corresponding direction, exacerbating stress concentration in that region. Simultaneously, the force from the cylinder is transmitted to the pin via the sleeve-contact point, further elevating stress levels in this area due to localized force concentration at the contact interface. Consequently, the high-stress zone connecting line aligns with the stress transmission path of the cylinder axis. Additionally, the low-stress zones exhibit a distinct directional complementary feature: their connecting direction is perpendicular to that of the high-stress zones, meaning the low-stress zones concentrate at the vertical ends of the pin. This forms a right-angle distribution pattern with the horizontal high-stress zones, perfectly matching the force flow diffusion path after the pin is subjected to force.

### 4.2. Discussion of the Pin Shaft 360° Rotation Results

The consistency between the average test values and simulation values is evaluated using a deviation index. To ensure uniform evaluation criteria, the deviation calculation process takes the measured test values as the reference benchmark. This quantifies the numerical difference between them. The calculation method is as follows [29]:

(2) $$\mathrm{Deviation}\;\left(\%\right)\;=|\frac{Experimental\;Value-Simulated\;Value}{\mathrm{Experimental}\;\mathrm{Value}}|\times \;100\%$$

**Figure 13:**
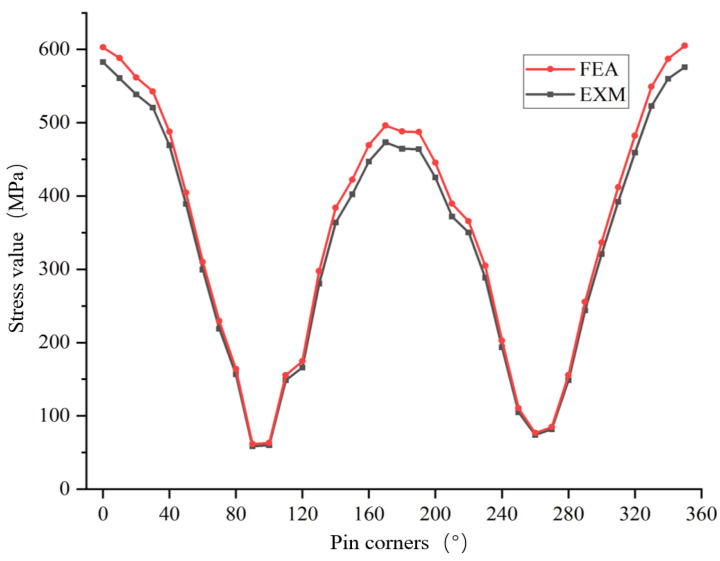
Comparison chart between test mean and calculated value.

**Figure 14:**
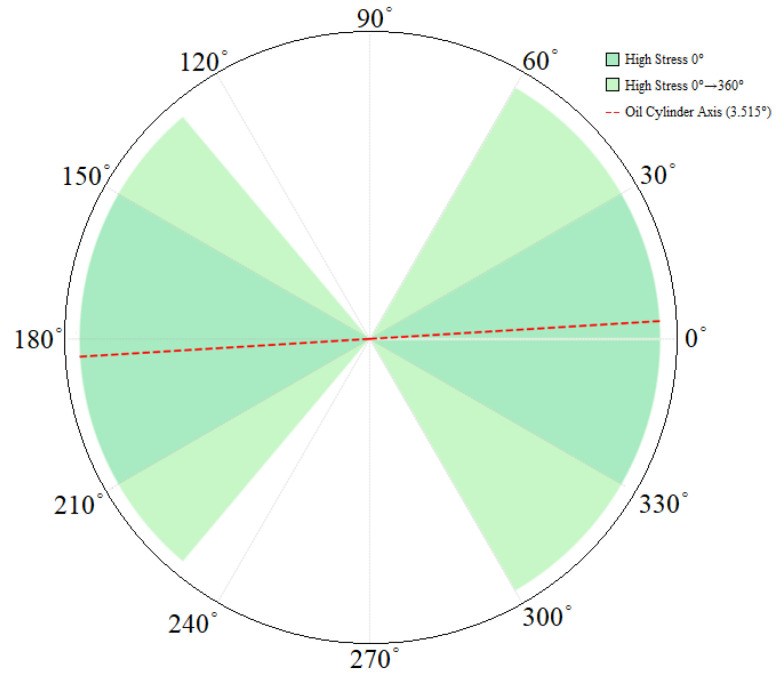
Distribution range of high-stress zones under the condition of 0 ° installation angle and 360 ° rotation of the pin shaft.

(1) As shown in Table 6, the deviation data corresponding to each angle (from 0° to 360°, with a 10° interval) indicate that the deviation between the average test values and simulation values ranges from 3.46% (at 0°) to 6.02% (at 130°). Within the entire angular range, the deviation does not exceed 6.1%, and the majority of deviations are concentrated between 4% and 5.5%. This indicates a low and relatively uniform overall deviation level. Combined with previous analysis of stress variation patterns, the stress variation trends at each angle between the test values and simulation values are fully consistent (both exhibit periodic fluctuations, with core low points and high-value intervals aligned). This further confirms the reasonableness of the deviation data. Considering the two characteristics of the deviation being controlled within a narrow range and showing a highly consistent variation trend, it can effectively verify the accuracy and reliability of the pin stress simulation model established in this study. This provides valid data support for subsequent pin structural adjustments and performance analysis.

(2) Plotting the stress test values and simulation values from 0° to 350° (in 10° intervals) as point-line diagrams (as shown in Figure 13), the distribution of data points and the curve fitting effect indicate that the stress values from both sources remain highly consistent at all angles, with deviations falling within a small range. This visually demonstrates the consistency between the test and simulation results. Additionally, as the pin rotation angle changes, the stress values exhibit a significant periodic fluctuation characteristic: stress reaches its peak at angles of 0°, 180°, and 360° (corresponding to 0°), and its trough at 90° and 270°, forming a “two peaks and two valleys” periodic distribution pattern. Further comparison with the high-stress zone distribution characteristics when the oil hole installation angle is 0° reveals that regardless of the pin’s rotation angle, the high-stress zones consistently concentrate near 0° and 180°. This indicates the stability of the periodic characteristics in the pin’s stress distribution across different rotation angles, with the high-stress zones remaining fixed. The essence of this phenomenon lies in the load transmission path of the cylinder axis direction: when the oil hole installation angle is 0°, the load transmission path from the cylinder axis directly acts on the pin’s horizontal plane, making the stress concentration effect in that direction more pronounced. Even if the pin rotates, as long as the spatial position of the cylinder axis remains unchanged, the relative relationship of the load transmission path to the pin’s geometric symmetry remains constant, ultimately resulting in the high-stress zones consistently concentrating near 0° and 180°, in line with the cylinder axis direction. This verifies the decisive role of the load transmission path in the pin’s stress distribution.

(3) By extracting the bolded numbers in Table 4 and Table 5 that represent high-stress states, the range of high-stress zones for the pin under a full 360° rotation was statistically determined and compared with the high-stress zones at a 0° installation angle. The results, as shown in Figure 14, reveal significant overlap between the high-stress zones at 0° and those occurring throughout the 0° to 360° rotation process, indicating the stability of the core distribution range of high-stress zones. Additionally, Figure 14 clearly shows that the cylinder axis (at an angle of 3.515°) forms a straight line span through the entire high-stress zone, with the high-stress zones remaining symmetrically concentrated around this axis regardless of the pin’s rotation angle. This further verifies the dominant role of the cylinder axis in the distribution of high-stress zones. Based on this analysis, to avoid stress concentration and potential failure risks associated with high-stress zones, the pin installation angle can be adjusted to the intervals [60° to 130°] and [230° to 300°]. These intervals avoid the high-stress concentration zones symmetric about the cylinder axis, effectively reducing the probability of structural failure due to stress concentration and providing crucial technical guidance for the installation and adjustment of pump truck pins.

(4) The FEA results indicate that there is significant tensile stress concentration at the edge of the lubricant hole of the pin, with a certain degree of shear stress also present around the hole. Macroscopic observation of the fracture surface shows that the crack initiation site is located at the edge of the lubricant hole; the initiation zone exhibits a flat cleavage surface (a tensile characteristic), while the propagation zone features an inclined shear lip (a shear characteristic). Microscopically, both cleavage steps and dimples are observed to coexist. Comprehensive analysis concludes that the primary failure mode of the pin is a composite fracture caused by the combined action of tensile and shear loads.

(5) In heavy-duty boom systems, pins serve as core force transmission hub components, with their load-bearing performance and structural reliability directly determining the overall service safety of the equipment, playing a crucial role in ensuring the long-term, efficient, and stable operation of heavy machinery. Typically, a large-scale heavy-duty machine integrates nearly a hundred pins, most of which suffer from excessive weight. This not only increases the total load on the boom system but may also indirectly affect the rationality of the mechanism’s force distribution. Therefore, after completing the precise adjustment of the pin oil hole installation angle (ensuring strict perpendicularity between the oil hole installation angle and the cylinder axis), initiating lightweight design research for the pins becomes a necessary step. Through lightweight design, the pins’ self-weight can be effectively reduced, thereby improving the force distribution of the entire boom mechanism and providing technical support for enhancing the mechanical performance and service life of heavy-duty boom systems.

## 5. Conclusions

In response to the frequent failure accidents of the pin shafts in concrete pump trucks during actual construction scenarios, this study first put forward targeted hypotheses regarding their failure mechanisms. Subsequently, with the aim of “validating these hypotheses and revealing the stress distribution patterns,” a synergistic analysis framework combining “experimental testing and numerical simulation” was established to systematically investigate the stress distribution issues of the pin shafts. The research focused on two core directions: first, the evolution characteristics of stress concentration under different installation angles; second, the spatial positional correlation between high-stress zones and the cylinder axis. Through the mutual validation of experimental data and simulation results, the following conclusions are provided:

(1) Breaking through the traditional “load-dominated” framework, a new failure mechanism of “installation angle-axis-line-stress” has been established. Traditional studies often attribute the pin shaft failures to excessive loads and material fatigue, neglecting the influence of installation parameters, making it difficult to explain “failure under low loads.” This study proposes a hypothesis for the first time: improper installation angle (the angle between the oil passage axis and the horizontal plane) may cause the oil hole to fall into the high-stress zone dominated by the cylinder axis. Through experimental and simulation validation, when the oil hole axis is parallel or nearly parallel to the cylinder axis, the stress concentration peak at the oil hole approaches the material’s yield strength, ultimately forming a causal chain: “installation angle deviation → oil hole enters the high-stress zone → stress exceeds limits → fatigue failure.” This fills the research gap on the impact of installation angles on the pin shaft failures.
(2) Constructing a “Dynamic Testing + Safety Factor Reverse Design” collaborative system to enhance the reliability of results. First, an innovative dynamic full-angle testing design is adopted, breaking the limitations of traditional fixed-angle static testing: by applying 360° stepwise loading to the pin shaft, the periodic characteristics of “angle-stress” are accurately captured, improving the engineering adaptability of the test data. Second, finite element safety factor reverse design is implemented: addressing the issue of traditional simulation ideal parameters overestimating strength, the simulation uses parameters without heat treatment under conditions where the tested pin shaft undergoes heat treatment beyond the yield strength. This approach leverages material performance deviations to retain safety factors, balancing analysis safety and model practicality.
(3) Quantifying the stability and periodic coordination of high-stress zones provides a basis for precise regulation. Traditional studies can only qualitatively determine that oil holes are prone to stress concentration, and fail to clarify their distribution range and variation patterns. Through refined analysis, this study reveals two core laws for the first time: First, the spatial stability law of high-stress zones confirms that the high-stress zones of the pin shaft do not shift with the rotation of the installation angle, always concentrating symmetrically around the cylinder axis (3.515°) in intervals such as [0° to 30°], providing quantitative support for avoiding high-stress zones. Second, the periodic coordination law between angles and stress captures the “dual-peak two-valley” periodic characteristics, with the period coordinating with the force flow path of the cylinder axis, breaking through the traditional understanding of “random stress fluctuations” and identifying low-stress safe intervals.
(4) Expanding the Application Scenarios from “Post-Failure Analysis” to “Full-Cycle Active Prevention”: Traditional studies often stop at failure cause analysis, offering general yet impractical recommendations. This research innovatively establishes a “Full-Cycle Prevention System”: On one hand, based on identified patterns, precise installation guidelines are proposed, adjusting the pin shaft installation angle to a safe zone perpendicular to the hydraulic cylinder axis. This significantly enhances the service life of the pin shaft structure and provides direct on-site guidance, addressing the “vagueness” issue of conventional suggestions. On the other hand, the entire technical chain is extended by integrating angle control with lightweight design, predictive maintenance of oil hole status, and automated sensing monitoring, forming a closed-loop system of “Design–Installation–Maintenance–Monitoring.” This provides a replicable paradigm for the long-term service of key components in similar construction machinery.

## Figures and Tables

**Table 1:** Arm structure parameters and geometric dimensions of the pin shaft table.

H (m)	*ω*_1_ (°)	*ω*_2_ (°)	*ω*_3_ (°)	*ω_oc_* (°)
12.2	60	47.13	120	60

**Table 2:** Geometric parameters of the pin shaft structure.

r_1_ (m)	r_2_ (m)	R_3_ (m)	L_1_ (m)	L_2_ (m)
0.055	0.02	0.004	0.254	0.025

**Table 4:** Statistical table of the pin shaft test mean values.

Pin Axis Angle (°)	Test Mean Value (MPa)	Pin Axis Angle (°)	Test Mean Value (MPa)
0	**583.09**	180	**465.82**
10	**561.04**	190	**465.19**
20	**537.51**	200	**427.12**
30	**523.09**	210	**374.57**
40	**472.26**	220	**352.01**
50	**390.25**	230	288.47
60	**299.38**	240	196.71
70	223.63	250	107.93
80	160.45	260	74.31
90	62.52	270	81.59
100	63.19	280	154.46
110	151.48	290	247.10
120	167.19	300	**323.37**
130	283.35	310	**395.10**
140	**365.44**	320	**458.76**
150	**404.96**	330	**526.76**
160	**446.75**	340	**560.47**
170	**478.15**	350	**577.81**

The bold black numbers represent the high-stress data where the stress value is ≥290 MPa.

**Table 5:** Statistical table of the pin shaft calculated values.

Pin Axis Angle (°)	Calculated Value (MPa)	Pin Axis Angle (°)	Calculated Value (MPa)
0	**602.82**	180	**347.9**
10	**588.17**	190	**487.28**
20	**561.65**	200	**445.56**
30	**542.6**	210	**359**
40	**487.58**	220	**365.49**
50	**404.51**	230	**304.85**
60	**310.04**	240	202.6
70	229.4	250	110.61
80	164.06	260	76.74
90	61.59	270	84.84
100	63.07	280	155.45
110	155.53	290	255.67
120	174.56	300	**336.65**
130	**297.42**	310	**411.79**
140	**384.02**	320	**482.25**
150	**322.36**	330	**549.1**
160	**469.44**	340	**587.15**
170	**495.97**	350	**604.89**

The bold black numbers represent the high-stress data where the stress value is ≥290 MPa.

**Table 6:** The pin shaft test mean vs. calculated value deviation.

Pin Axis Angle (°)	Deviation	Pin Axis Angle (°)	Deviation
0	3.46%	180	5.04%
10	4.89%	190	5.04%
20	4.29%	200	4.77%
30	4.25%	210	4.70%
40	3.92%	220	4.36%
50	3.91%	230	5.63%
60	3.51%	240	4.72%
70	4.78%	250	5.27%
80	4.60%	260	3.81%
90	5.26%	270	3.98%
100	5.27%	280	4.56%
110	4.56%	290	4.83%
120	5.26%	300	4.94%
130	6.02%	310	4.99%
140	5.56%	320	5.04%
150	5.00%	330	5.06%
160	5.03%	340	4.89%
170	4.82%	350	5.08%

## Data Availability

The data that support the findings of this study are available from the corresponding author upon reasonable request.
